# The Effect of Adjuvant Zinc Therapy on Recovery from Pneumonia in Hospitalized Children: A Double-Blind Randomized Controlled Trial

**DOI:** 10.1155/2014/694193

**Published:** 2014-05-12

**Authors:** Mohammad Javad Qasemzadeh, Mahdi Fathi, Maryam Tashvighi, Mohammad Gharehbeglou, Soheila Yadollah-Damavandi, Yekta Parsa, Ebrahim Rahimi

**Affiliations:** ^1^Department of Medicine, Islamic Azad University, Qom Branch, Qom, Iran; ^2^Students' Research Committee, Islamic Azad University, Tehran Medical Branch, Tehran, Iran; ^3^School of Public Heath, Department of Epidemiology, Shahid Beheshti University of Medical Sciences, Tehran, Iran

## Abstract

*Objectives*. Pneumonia is one of the common mortality causes in young children. Some studies have shown beneficial effect of zinc supplements on treatment of pneumonia. The present study aimed to investigate the effects of short courses of zinc administration on recovery from this disease in hospitalized children. *Methods*. In a parallel Double-Blind Randomized Controlled Trial at Ayatollah Golpaygani Hospital in Qom, 120 children aged 3–60 months with pneumonia were randomly assigned 1 : 1 to receive zinc or placebo (5 mL every 12 hours) along with the common antibiotic treatments until discharge. Primary outcome was recovery from pneumonia which included the incidence and resolving clinical symptoms and duration of hospitalization. *Results*. The difference between two groups in all clinical symptoms at admittance and the variables affecting the disease such as age and sex were not statistically significant (*P* < 0.05) at baseline. Compared to the placebo group, the treatment group showed a statistically significant decrease in duration of clinical symptoms (*P* = 0.044) and hospitalization (*P* = 0.004). *Conclusions*. Supplemental administration of zinc can expedite the healing process and results in faster resolution of clinical symptoms in children with pneumonia. In general, zinc administration, along with common antibiotic treatments, is recommended in this group of children. It can also reduce the drug resistance caused by multiple antibiotic therapies. This trial is approved by Medical Ethic Committee of Islamic Azad University in Iran (ID Number: 8579622-Q). This study is also registered in AEARCTR (The American Economic Association's Registry for Randomized Controlled Trials). This trial is registered with RCT ID: AEARCTR-0000187.

## 1. Introduction 

Acute lower respiratory tract infection is one of the most important and common diseases among children, which is accompanied by high mortality rate, especially in young children. This infection is the most important cause of mortality among children under 5 in developing countries, accounting for nearly one-third of the cases [1–4].

Pneumonia is one of the most common implications of lower respiratory tract involvement. The World Health Organization estimates that of approximately 4 million annual deaths due to pneumonia, half of the cases occur in children less than 1 year of age [2, 3, 5].

On the other hand, malnutrition plays a significant role in the increased prevalence, severity, and prognosis of pneumonia, especially among children [3].

Zinc and iron deficiency is one of the most common nutritional problems in Iran and many developed countries. According to statistics, about 50% of the common nutritional problems are due to a combined deficiency of the two elements, though the beneficial role of zinc compared to iron has been forgotten in Iran [6].

Zinc is an essential nutritional element, with a broad spectrum of biological activities in humans. This element plays an important and vital role in the physical development of digestive and immune systems. Zinc deficiency in children can cause stunted growth and increased incidence of infections (pneumonia, gastroenteritis) through weakening the immune system and changing neural and behavioral actions [1, 7].

Numerous studies on therapeutic and prophylactic effects of zinc administration in infectious diseases indicate that administration of zinc compounds significantly reduces the incidence of gastroenteritis and pneumonia, and its deficiency could cause immune system deficiencies and increase the risk of serious infectious diseases such as diarrhea and malaria [1, 8–10].

Another study showed that serum zinc level in children with pneumonia and gastroenteritis was lower than in those of the same age [11].

It should be noted that inadequate intakes of zinc in the diet were the main cause of zinc deficiency. The annual report of the World Health Organization in 2003 has emphasized on the importance of adding zinc as a food supplement to the diet. The clinical symptoms of zinc deficiency during early childhood include acute or chronic diarrhea accompanied by malnutrition, psychiatric disorders, and behavioral problems. A chronic zinc deficiency could cause alopecia, stunted growth, skin lesions, and common childhood infections such as pneumonia [12].

Zinc supplements can prevent and decrease the incidence of pneumonia. It can also shorten diarrhea episodes and resolve them [10, 13, 14].

The World Health Organization (WHO) and the United Nations Children's Fund (UNICEF) recommend that the children living in developing countries should take zinc supplement for 10 to 12 days as follows: 10 mg daily for infants younger than 6 months and 20 mg daily for infants older than 6 months. The purpose of this treatment is to reduce the severity of acute diarrhea episodes and hasten recovery from severe pneumonia in developing countries [7].

Given what was previously mentioned about the beneficial effects of administrating zinc compounds for prevention or treatment of pneumonia among children, and knowing that only a few studies have been conducted in this field, especially in Iran [15], the present study aimed to determine the effect of zinc on clinical course of 3 to 60-month children hospitalized due to pneumonia. It was assumed that this element (zinc) was effective in resolving clinical symptoms and duration of hospitalization.

## 2. Materials and Methods

This study was a parallel, double-blind, randomized controlled clinical trial conducted on 120 children aged 3–60 months suffering from pneumonia, after obtaining permission from the University Research Council and Ethics Committee. The participants were all patients hospitalized at Ayatollah Golpaygani Hospital in Qom, Iran, from June 2012 to June 2013. Moreover, the children's parents completed and signed the informed written consent forms for conducting the study and the relevant experiments before entering the study.

The participants were randomly assigned, following simple randomization procedures (1 : 1), to receive zinc or placebo (5 mL every 12 hours) along with the common antibiotic treatments until discharge. Participants and their parents and those assessing the outcomes were blinded to group assignment.

Patient assigned to placebo received the same solution as zinc supplement syrup but without zinc.

Primary outcome was recovery from pneumonia which included the incidence and remission of clinical symptoms and duration of hospitalization.

Diagnostic criteria of pneumonia and signs and symptoms were recorded according to Nelson Textbook of Pediatrics [7].

Family history of respiratory diseases, infections leading to hospitalization, duration of symptoms and taking medication at home, and influenza vaccination in the participants' families were recorded. Moreover, symptoms such as cough, fever, tachypnea, tachycardia, respiratory distress at admittance, and pleural effusion and bronchopneumonia on chest X-ray were evaluated. Then disease symptoms, duration of hospitalization, and duration of the resolution of clinical symptoms were investigated.

The children taking zinc compounds, those suffering from severe malnutrition, and those with the symptoms of gastroenteritis, and other diseases, were excluded from the study.

The data were analyzed by running the Chi-square and Fisher's tests using SPSS 16. The level of significance was considered 0.05.

## 3. Results

A total of 120 pneumonia patients were hospitalized from June 2012 to June 2013; 60 were randomized to treatment and 60 to placebo. None of them loss and exclude after randomization.

The baseline characteristics were well balanced in the 2 study groups. As shown in Table 1, there was no statistically significant difference between the two groups in terms of the age and sex variables.

The family history of respiratory diseases in the treatment and control groups was 18.3 and 13.3, respectively. About the administration of influenza vaccine in family, 3.3% of the subjects in the treatment group had a history of injection in the family, while the control group had no history of vaccination. In the treatment group, the rate of infection leading to hospitalization as well as pneumonia (for both variables) was estimated as 30%. In the control group, the rate of corresponding infections was 43.3% and 40%, respectively. There was no statistically significant difference between all these proportions.

In the treatment group, the incidence of symptoms such as cough, fever, tachypnea, tachycardia, and respiratory distress at admittance was reported as 96.7, 36.7, 10, 6.7, and 15%, respectively. In the control group, this incidence was reported as 100, 25, 8.3, 3.3, and 16.7%, respectively. There was no statistically significant difference between these proportions in both groups.

Moreover, there was no significant difference between the evaluation of chest X-ray in the two groups in terms of pleural effusion and bronchopneumonia. The prevalence of radiological findings in the treatment group was 50% and 26.7%, respectively, and the prevalence of corresponding values in the control group was 48.3% and 25%, respectively.

Table 2 shows the duration of symptoms and taking medication at home as well as the duration of hospitalization and remission of clinical symptoms. As can be seen, there was a significant difference between the two groups in terms of the duration of symptoms and hospitalization.

## 4. Discussion

The present study aimed to evaluate the effect of zinc on the clinical course of pneumonia in 3 to 60-month-old children hospitalized in pediatric wards.

Implementation of a random allocation might be a possible reason for the fact that in this trial, there was no statistically significant difference between the two groups in terms of some factors affecting the disease such as age, sex, family history of respiratory infections, and infections leading to hospitalization. Similar symptoms such as cough, fever, tachypnea, tachycardia, and respiratory distress in the above-mentioned groups at admittance and before the intervention indicate that the severity of the disease has been almost the same. Therefore, the major difference between the children in the treatment group can be found in the element of zinc, which is administered along with the standard antimicrobial therapy for pneumonia.

In this study and compared to the comparison group, a significant decrease was found in the duration of hospitalization and recovery from pneumonia symptoms in zinc-receiving children. This indicates the effect of zinc therapy and a change in the clinical course of pneumonia among the children under investigation. This finding is consistent with the results of most studies in this field [5, 16–18], some of which are mentioned below.

Brooks et al. reported that prescription of zinc in 2 to 23-month children suffering from severe pneumonia leads to significant reduction in the severity of tachypnea, anorexia, restlessness, and duration of hospitalization [5].

A similar study in India (2007) on 299 children aged 2–23 months and hospitalized due to severe pneumonia showed that, compared to the comparison group, disease symptoms were improved faster and the duration of hospitalization decreased significantly in the zinc-receiving patients [17].

In another similar study in India that was conducted on 153 children aged 2–24 months, who were hospitalized due to acute lower respiratory infection and divided into two groups (one taking 10 mg of zinc plus vitamin A daily, and the other taking placebo plus vitamin A), it was shown that the recovery time was significantly faster in the treatment group than in the control group. Overall, zinc therapy can reduce the duration of symptoms and acute clinical condition [18]. This finding is consistent with the findings of our study.

Moreover, another field trial with similar implementation in India (2003), however, on 2482 healthy children aged 6–30 months, showed that the prevalence of pneumonia was lower in the treatment group than in the control group. This indicates that in addition to reducing the duration of symptoms and expediting the healing process in patients suffering from pneumonia, zinc can also prevent this disease [16]. In a study at the University of Mashhad on 200 early school age children (2009), it was found that, compared to the control group, the number of cold attacks reduced in the zinc-treated group. This shows that zinc can be useful in the prevention of respiratory diseases among children through improving their nutritional status [14].

## 5. Conclusion

According to the results of the present study and comparing them with other similar studies in this field, it can be inferred that zinc can hasten the recovery from pneumonia and quickly resolve its symptoms in children suffering from this disease. Overall, using zinc along with antibiotic therapies is recommended in this group of children. Zinc therapy can also reduce the drug resistance caused by multiple antibiotic therapies. Hence, in order to improve the clinical course and duration of symptoms, it is recommended to administer zinc supplementation to the children with suspected respiratory symptoms on their arrival at the hospital. We recommended that further studies with larger sample sizes would be useful in confirming the results of this study and reaching a conclusive opinion in this field.

## Figures and Tables

**Table 1:** Frequency distribution of the study population by age and sex^↑^.

	Status	Treatment group	Control group	*P* value*
Age	<6 months	23 (38.34)	25 (41.67)	0.929
	Six months–2 years	32 (53.34)	30 (50)	
	2–5 years	5 (8.34)	5 (8.34)	

Sex	Male	35 (58.34)	34 (56.67)	0.853
	Female	25 (41.67)	26 (43.34)	

^↑^Both groups included 60 members. The corresponding values in the table are the numbers (percentage).

*Chi-square: *α* = 0.05.

**Table 2:** Characteristics of the study population according to the duration of symptoms and taking medication at home as well as the duration of symptoms and hospitalization in the comparison groups^↑^.

	Treatment group			Control group			*P* value*
	<2 days	2–5 days	>5 days	<2 days	2–5 days	>5 days	
Duration of symptoms at home	11 (18.3)	23 (38.3)	26 (43.34)	13 (21.7)	27 (45)	20 (33.3)	0.53
Duration of taking medication at home	19 (31.7)	24 (40)	17 (28.3)	21 (35)	19 (31.7)	20 (33.3)	0.63
Duration of symptoms at hospital	30 (50)	22 (36.7)	8 (13.3)	25 (41.7)	33 (55)	2 (3.3)	0.044
Duration of hospitalization	12 (20)	36 (60)	12 (20)	2 (3.3)	34 (56.7)	24 (40)	0.004

^↑^Both groups included 60 members. The corresponding values in the table are number (percentage).

*Chi-square: *α* = 0.05.
